# Social and physiological stress elicit divergent psycho-physiological dynamics and motor cortex activation

**DOI:** 10.3389/fpsyg.2026.1760772

**Published:** 2026-03-05

**Authors:** Maria Koriakina, Mikhail Lukov, Uliana Nikishkina, Aleksandr Kirsanov, Ekaterina Dmitrieva, Evgeny Blagovechtchenski

**Affiliations:** Laboratory of Behavioural Neurodynamics, Saint-Petersburg State University, Saint-Petersburg, Russia

**Keywords:** autonomic nervous system, blood pressure, Cold Pressor Test, corticospinal excitability, heartrate variability, motor evoked potentials, psychophysiology, stress response

## Abstract

The investigation of question whether a characteristic stress response pattern can be assessed through the level of motor cortex activation. This study compared the distinct effects of socio-psychological (Trier Social Stress Test, TSST) and physiological (Cold Pressor Test, CPT) stressors on the dynamics on heart rate (HR), blood pressure (BP), and subjective emotional state dynamics. The study had two primary objectives: (1) to assess the differential effects of these stressors by comparing the temporal dynamics of both physiological and psychological parameters; and (2) to estimate the relationship between corticospinal excitability and peripheral autonomic responses during stress. Motor evoked potentials from first dorsal interosseous were induced by stimulation of the primary motor cortex using transcranial magnetic stimulation (TMS), the temporal changes in all measures were analyzed at three time points: pre-stress baseline, immediately post-stress, and after a 15-min recovery period. Psychological stress induced a sustained increase in tension, a decrease in positive affect, and a prolonged peripheral activation (e.g., in HR, systolic BP). In contrast, physiological stress elicited a transient reaction, followed by rapid normalization and even a subsequent decrease in some psychophysiological indices. Analysis of subjective psychological reports showed that the TSST in-creased self-reported tension and decreased positive affect, in contrast to the CPT, which led to a reduction in tension and anxious-depressive emotions. TMS-measured cortico-spinal excitability showed a direct covariation with heart rate, indicating a tight functional coupling between the motor and autonomic nervous systems during physiological stress.

## 1 Introduction

The Research in the psychophysiology of stress continues to reveal the complex interplay between the psychological and physiological components of the stress response. Despite this progress, fundamental questions remain about how different types of stressors impact the dynamics and concordance of physiological and subjective parameters. The existing literature is characterized by inconsistent methodology and conflicting data regarding the relationship between objective autonomic markers (e.g., heart rate and blood pressure) and subjective emotional states (Cameron, 2001; Campbell and Ehlert, 2012). Consequently, a critical and unresolved challenge is determining whether central nervous system components of the stress response are stressor-specific, as distinct from peripheral autonomic changes. For example, autonomic arousal may manifest similarly across stressors, but central markers, such as motor cortical excitability, may reveal stressor-specific activation patterns (Cao et al., 2021). These patterns offer a more nuanced signature of the stress response. Of particular scientific and medical interest are the temporal dynamics of cardiovascular parameters under different types of stress exposure and the potential dissociations between objective physiological markers of stress and subjective emotional state assessments (Sumińska and Rynkiewicz, 2025).

The Numerous studies confirm that psychological and physiological stressors activate distinct physiological adaptation mechanisms (Skoluda et al., 2015). The key difference lies in the nature of the threat. Psychological stressors represent perceived impacts that require complex cognitive-evaluative processes and engage higher-order brain regions, such as the prefrontal cortex and limbic system. These regions then trigger a robust neuroendocrine response (Sarmiento et al., 2024). This response involves the sympathoadrenal medullary (SAM) system and the hypothalamic–pituitary–adrenal (HPA) axis, leading to significant cortisol release. In contrast, physiological stressors (e.g., the Cold Pressor Test or exercise) present a direct physical challenge to homeostasis. They primarily elicit a strong, direct sympathetic outflow to regulate bodily functions and involve the HPA axis to a lesser extent, often with a complex, rapid interplay of sympathetic and parasympathetic influences (Herman et al., 2016).

The term “autonomic nervous system” (ANS) is also used because it accurately describes the part of the nervous system responsible for involuntarily controlling physiological parameters, such as blood pressure and heart rate, which are central to our investigation of stress.

There are different ways of stress induction. Most commonly used stress-induction protocols are: The Cold Pressor Test (CPT) and the Trier Social Stress Test (TSST; Allen et al., 2014; Fanninger et al., 2023; Kirschbaum et al., 1993; McRae et al., 2006). While both are standardized methods for inducing stress, they elicit different types of stress responses. The CPT is a physiological stress test during which a participant immerses their hand in ice-cold water (0–4 °C) for a specified duration (e.g., 1–3 min) (Fanninger et al., 2023). In contrast, the TSST is a standardized laboratory protocol designed to induce moderate socio-evaluative threat. It consists of three stages: preparing a speech, delivering it before a panel of evaluators, and performing a mental arithmetic task (Allen et al., 2017).

Existing data on the correlation between subjective stress experiences and objective physiological indicators across different types of stress are notably inconsistent. Birkett (2011) demonstrated that the TSST elicits a significant increase in cortisol and alpha-amylase levels, confirming its efficacy as a method for inducing social stress (Birkett, 2011). The review by Sumińska et al. indicated that the TSST provokes the most pronounced and intense physiological shifts, including a sharp 50–70% rise in cortisol levels, accompanied by a significant increase in heart rate of 20–30 beats per minute. Moreover, this stressor activates the entire hypothalamic–pituitary–adrenal (HPA) axis and leads to a substantial elevation of systolic blood pressure by 15–20 mmHg, reflecting a robust autonomic response to social threat (Sumińska, 2022).

Findings by Skoluda et al. (2015) revealed significant differences in response patterns to different stress types. The TSST was associated with the highest level of subjective stress perception (as measured by a visual analog scale), with scores increasing by 65–75% from baseline. This test also induced the most pronounced HPA axis activation, manifested by an average increase in salivary cortisol concentration of 2.8 nmol/L (a 145% increase from baseline). In contrast, the CPT primarily elicited a sympathetic-adrenal response, characterized by an increase in heart rate by 22 ± 3 bpm and systolic blood pressure by 18 ± 2 mmHg, while the cortisol response was minimal (an increase of only 0.7 nmol/L) (Skoluda et al., 2015).

However, a recent systematic review by Fanninger et al. (2023) provided a critical analysis of the methodological aspects of CPT application in pain and stress research. The authors identified substantial variability in test protocols, leading to significant ambiguity in the obtained results. Particular attention was paid to three key parameters: water temperature, immersion depth, and exposure duration (Fanninger et al., 2023).

Thus, despite the existence of standardized stress-induction methods, unresolved questions remain concerning: (1) the degree of concordance between psychological and physiological reactions, (2) the factors contributing to the variability of the stress response, and (3) the methodological limitations of existing approaches (Fanninger et al., 2023; Mauss et al., 2005).

The present study addressed these gaps by implementing a comparative design that directly contrasts a psychosocial and a physiological stressor within a unified methodological framework. This approach allows for a direct investigation of the stressor-specific concordance between subjective emotional reports, ANS activity (heart rate, blood pressure), and a novel central nervous system marker—motor cortex excitability. By employing this multi-system assessment, we aimed to elucidate the sources of response variability and provide a more integrated signature of the stress response.

Frequent discrepancies between subjective and objective measures complicate the interpretation of stress reactivity and resilience (Mauss et al., 2005). One potential source of this inconsistency is the predominant focus on peripheral or isolated central measures. In order to capture an individual’s characteristic pattern of stress response, a quantifiable, integrated approach that considers the interplay between central and peripheral systems is necessary.

The stress response engages a broad network of brain regions and systems. Although the sensory and limbic systems, which process the emotional aspects of stress, have been the primary focus of research, recent studies have highlighted the substantial role of the motor system in the stress response cascade (Salo et al., 2019). The predominant cognitive framework largely conceptualizes stress in terms of altered cognitive reactions, overlooking the fact that the systemic response of the organism inherently requires the engagement of the motor system. According to the classical “Sherrington’s funnel” model, the motor system is the final stage of the reaction to a stimulus because it enables interaction with the external environment (Grillner and El Manira, 2020; Kandel et al., 2021). Thus, these systems are inextricably linked, and this link is supported by a growing number of evidence (see further in the text).

The primary motor cortex (M1) plays a pivotal role within the motor system, being fundamentally associated with the control of precise voluntary movements (Stinear et al., 2009). These movements are distinguished from automated motor programs and the operations of the ANS (Lemon, 2008). This distinction raises a critical question: How are the effects of stress reflected in the activity of the motor cortex? We propose measuring the excitability of the primary motor cortex as an integrated biomarker that captures the central-peripheral coordination as a part of adaptive stress response.

The primary motor cortex receives dense projections from limbic structures such as the amygdala and the anterior cingulate cortex (Rolls, 2019). These structures are central to threat processing and stress regulation (Sheena et al., 2021). This neuroanatomical substrate enables the rapid influence of emotional state on motor readiness, a phenomenon supported by clinical observations. For example, stress and anxiety can exacerbate motor symptoms in disorders such as Tourette syndrome (Godar and Bortolato, 2017) and induce tremors in healthy individuals (Louis, 2014). Furthermore, neuroimaging studies consistently show that acute psychosocial stress activates M1 alongside classic limbic regions (Pruessner et al., 2008; Vanhollebeke et al., 2022), suggesting its integral role in orchestrating the stress response. However, the precise dynamics of how stress alters the fundamental neurophysiological property of primary motor cortical excitability and how these changes are linked to autonomic outflow remain unclear. Given its key role in motor output and functional connection to stress networks, M1 offers a suitable area for investigating this engagement. Therefore, quantifying its excitability via motor-evoked potentials (MEPs) induced by transcranial magnetic stimulation (TMS) is a targeted approach to probing a key node in the stress-processing network, not merely a methodological convenience (Dedovic et al., 2009). This provides a direct window into the dynamics of the cortical motor system (Hallett, 2007). TMS is a unique neurophysiological tool and the only noninvasive method capable of providing a quantitative, direct measure of the net excitatory/inhibitory balance in the human cortex. This has been most consistently demonstrated for the excitability of the primary motor cortex (M1) and the integrity of corticospinal pathways (Hallett, 2007).

Furthermore, advanced analytical methods now allow for robust validation of heart-brain interactions through heartbeat-evoked potentials (HEPs) (Al et al., 2020). Importantly, HEPs reflect the processing of visceral afferent signals, and their amplitude is modulated by attentional focus on internal states and autonomic arousal levels (Al et al., 2023; Steinfath et al., 2025). This makes HEPs a particularly relevant measure of stress because they potentially capture the brain’s interpretation of bodily arousal during threat perception. Thus, they offer a unique electrophysiological window into the subjective experience of stress that complements objective ANS and cortical excitability measures (Lu et al., 2022). These studies showed that the activity of the nervous system is reflected in heart activity and that there are reciprocal relationships between the two systems. Of particular interest were the correlations between ANS and central nervous system (CNS) parameters.

The present study also assessed whether primary motor cortical excitability could serve as a biomarker that co-varies with key ANS reaction, such as blood pressure, as well as with subjective psychological states, across different stress paradigms. This would provide an integrated neurophysiological and psychological signature of the stress response. Although autonomic states such as cardiac phase can directly modulate cortical excitability (Al et al., 2023), it is unclear whether motor cortex reactivity could serve as a more sensitive discriminator of stressor types than autonomic indices alone. For example, physiological changes (e.g., HR and BP) may be similar across social and physiological stress. However, changes in MEPs may reveal distinct, stressor-specific patterns of central activation. Methodological variability across stress tests and the prevailing focus on objective (e.g., HR and BP) or subjective (e.g., psychological metrics) measures in isolation limits our understanding of the integrated, system-wide stress response.

The aim of the present study was:

(1) To identify the differential effects of the CPT and the TSST by conducting a thorough evaluation of physiological (e.g., blood pressure, heart rate) and psychological parameters. Particular emphasis was placed on analyzing the temporal dynamics of the stress response and the concordance between objective and subjective measures.
(2) To investigate the relationship between central nervous system activity (as indexed by motor cortex excitability) and peripheral autonomic responses under stress, by assessing the correlation between MEP elicited by TMS on M1 dynamics and cardiovascular changes.

The relevance of this study is driven by the need to address an existing methodological gap. Many studies on the CPT and TSST focus on either physiological correlates or subjective experiences, hindering a comprehensive assessment (Fanninger et al., 2023). Additionally, inconsistent findings regarding the concordance between objective and subjective stress markers and a lack of research directly comparing the impact of these stressors on these two types of responses limit our understanding of the plasticity of stress-reactive systems (Allen et al., 2014; Hellhammer et al., 2009).

Furthermore, the role of the motor cortex as an interface between cognitive and motor processes in the integrated stress response is not well understood. Chronic stress leads to reduced dendritic spine density, altered synaptic plasticity, and glial remodeling in the motor cortex—all of which are hallmarks of disrupted information processing and learning at the cellular and circuit levels (Gellner et al., 2022). Thus, assessing motor cortex excitability using TMS provides a unique, objective window into the state of cortical networks during stress, enabling the investigation of these central markers of stress. Focusing on motor cortex activation during stress improves our understanding of the mechanisms underlying motor symptoms, neuroplasticity, and stress vulnerability. This knowledge is crucial for translational neuroscience and clinical care.

Novelty: First, this work provides a comprehensive, comparative analysis of response dynamics for different types of stress under strictly controlled conditions. This enables the identification of specific adaptive mechanisms. Second, we used TMS as a diagnostic probe to estimate the relationship between motor system excitability and ANS activity during stress. Integrating the analysis of the temporal dynamics of psychophysiological parameters and MEPs provides a comprehensive view of the stress response and demonstrates the interaction between central and peripheral processes.

## 2 Methods

### 2.1 Participants

The study included 60 participants aged 18–25, who were divided into three groups: Group 1 consisted of 15 participants (6 males, 9 females) with a mean age of 20.1 years (SD = 2.3). Group 2 consisted of 15 participants (6 males, 9 females) with a mean age of 23.1 years (SD = 3.3). Group 3 (CPT with TMS measures) consisted of 15 participants (6 males, 9 females) with a mean age of 22.3 years (SD = 2.8). Participants were recruited from the local university student population via online advertisements. Exclusion criteria comprised any major neurological, psychiatric, or cardiovascular diagnosis, as well as the regular use of psychoactive or hormonal medication.

### 2.2 Experimental design

#### 2.2.1 Stress induction protocols

According to the experimental design, three groups of participants were exposed to different types of stress, both socio-psychological and physiological. The two tests aimed to study how induced stress affects recorded physiological parameters and subjective assessments of emotional state.

(a) The Trier Social Stress Test was administered according to the protocol outlined by Kirschbaum et al. (1993) and supplemented with physiological measurements. The protocol was adapted from the original TSST, with the pre-test rest period shortened to 5 min to align with current clinical guidelines for cardiovascular parameter stabilization prior to measurement. After a five-minute rest period, the participant’s blood pressure was measured, and he or she was asked to complete psychodiagnostics questionnaires assessing his or her current emotional state (see Section 3 in Methods). Then, the participant was instructed to prepare a 5-min self-presentation speech. During the preparation phase, they were given paper and a pencil to take notes, which were collected before the speech commenced. The speech commenced immediately after preparation and lasted 5 min. It was delivered in front of a panel of three individuals introduced as evaluators of verbal and nonverbal behavior based on predefined criteria. The evaluators maintained neutral facial expressions throughout the presentation and ensured that the participant spoke for the full 5 min. Next, the participant completed a five-minute mental arithmetic task, subtracting 13 from 1,022 step by step as quickly and accurately as possible. Each time an error was made, the panel interrupted the procedure and instructed the participant to start again from 1,022. After completing the arithmetic task, blood pressure was measured again, and emotional state was assessed using questionnaires. Then, a debriefing session was conducted to explain the artificial nature of the stressful situation. After a 15-min recovery period, timed from the completion of the arithmetic task, a final resting blood pressure measurement was taken.
(b) The Cold Pressor Test was administered according to the procedures outlined by Mitchell et al. (2004), including the recording of physiological parameters. After a five-minute rest period, the participant’s blood pressure was measured, and they were asked to complete psychodiagnostics questionnaires reflecting their current emotional state (as described in the TSST procedure above). Then, the participant moved to a sound- and light-attenuated chamber where they were seated in an adjustable chair. Upon the experimenter’s command, the participant immersed their left hand up to the wrist in a reservoir of cold water maintained at 4 °C. Participants could withdraw their hand temporarily if the pain became too intense. The exposure period lasted 3 min, and physiological indicators were recorded continuously. Immediately after the 3-min exposure period ended and the participant removed their hand from the water, blood pressure was measured again and the psychological questionnaires were completed. Fifteen minutes after the end of the exposure period, final measurements of blood pressure and a self-assessment of emotional state identical to the two preceding assessments were performed.

#### 2.2.2 Assessment of psychological state

**Figure 1:**
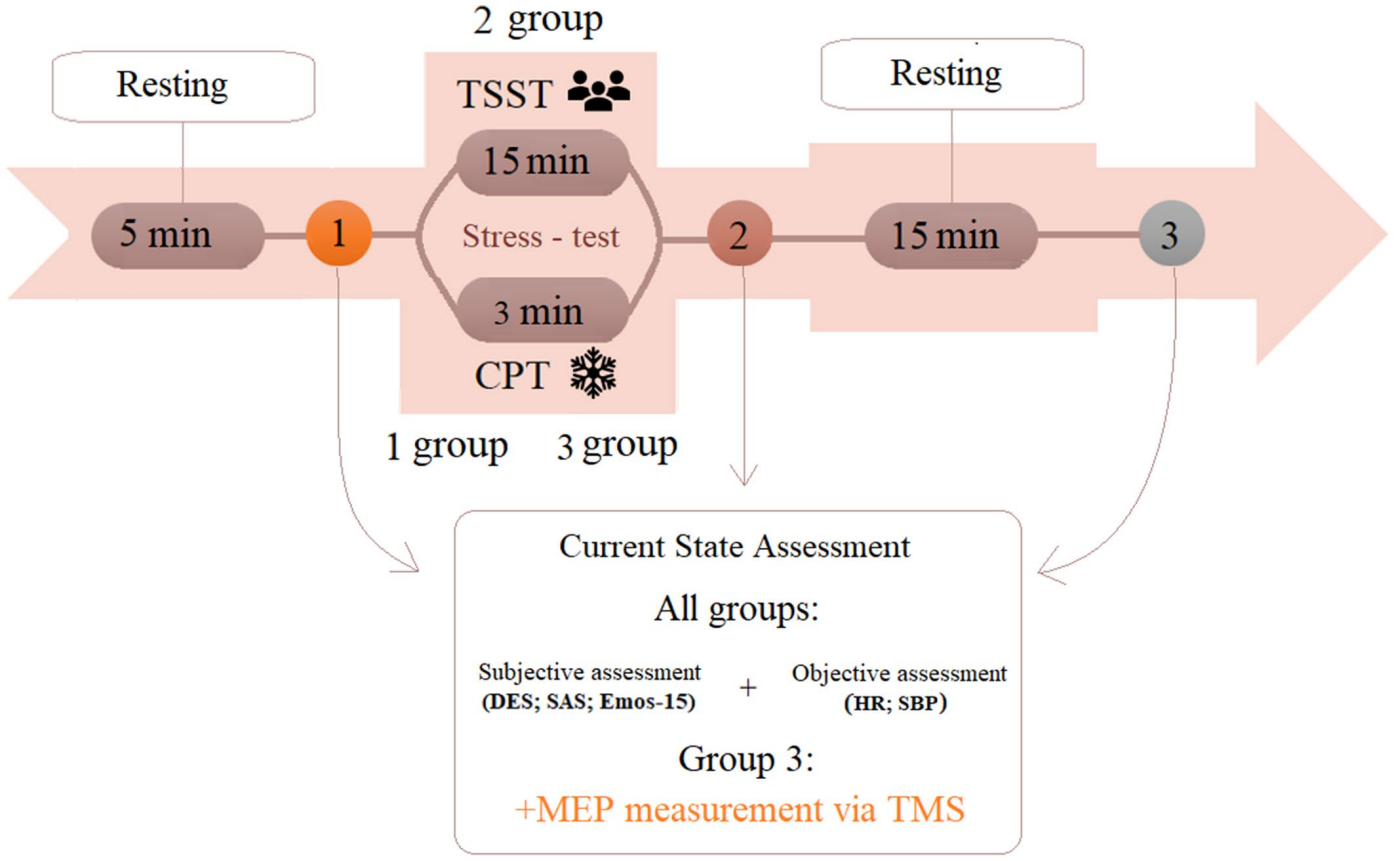
Experimental protocol for the induction of psychological and physiological stress types. TSST, Trier Social Stress Test; CPT, Cold Pressor Test; DES, differential emotions scale; SAS, self-rating anxiety scale; Emos-15, core affect scale; HR, heart rate; SBP, systolic blood pressure; MEP, motor evoked potentials, TMS, transcranial magnetic stimulation.

The psychological state was assessed using three validated self-report scales administered at three time points: before stress induction, immediately after, and after the 15-min recovery period (see Figure 1).

(a) The Differential Emotions Scale (DES; Izard, 2013)

This scale was used to assess the current emotional state. It consists of 30 adjectives reflecting the intensity of 10 fundamental emotions: interest, joy, surprise, pride, anger, disgust, contempt, fear, shame, and sadness. Each item is rated on a 5-point scale (“0—not at all” to “5—very strongly”). Scoring yields indices for positive and negative emotions and anxiety, reflecting the respondent’s current emotional state (Izard, 2013).

(b) Zung Self-Rating Anxiety Scale (SAS; Zung, 1971)

This scale measures the level of anxiety. It comprises 20 statements reflecting anxiety manifestations across four domains. A modified version of the scale was used in the experiment to assess current anxiety levels. Responses are coded from 1 to 4, with five items being reverse-scored. The raw sum is converted into an anxiety index ranging from 20 to 80, which is classified as minimal, moderate, or marked (Zung, 1971).

(c) The Emos-15 Core Affect Self-Report Scale (Lyusin, 2019)

This scale was used to diagnose the current emotional state and monitor emotional background. It measures core affect, which is an elementary, consciously accessible affective feeling that is non-object-oriented and represents the central component of any emotional phenomenon (Russell, 2003). In the Emos-15 scale, core affect is conceptualized using a dimensional approach and measured along three axes: positive emotions with high activation, negative emotions with low activation, and tension. Participants were asked to rate 15 emotion names on a five-point scale ranging from “does not apply at all” to “completely applies.” Responses are coded from 1 to 5, so the minimum possible score for each subscale is 5 and the maximum is 25 (Lyusin, 2019).

#### 2.2.3 Assessment of physiological parameters

The assessment of physiological indicators involved recording two parameters: SBP and HR. These were measured using an automatic blood pressure monitor under standardized conditions. Blood pressure was measured using an automatic oscillometric monitor (LD-521А, China). SBP served as an indicator of the cardiovascular system’s response to stress exposure. Meanwhile, HR was considered an objective marker of sympathetic nervous system activation and stress reaction intensity.

The measurement protocol involved three recordings of the physiological parameters (see Figure 1).

- Baseline measurements were taken after a five-minute adaptation period at rest to minimize the influence of external factors on physiological status.
- Post-stress measurements were taken immediately after the stress induction procedure ended to assess the acute stress response.
- A control measurement was conducted 15 min after the end of the experimental procedure to analyze the recovery dynamics.

#### 2.2.4 Transcranial magnetic stimulation (TMS) protocol

Cortical excitability was assessed by measuring MEPs, which were elicited by single-pulse TMS. We used a TMS system comprising a MagPro (X100, Magventure, Denmark) magnetic stimulator and a Localite TMS Navigator navigation (Localite, Germany) system with a 70-mm-diameter figure-eight coil (B-60, Magventure, Denmark). The coil was positioned over the primary motor cortex (M1) of the dominant hand area (left hemisphere), tangentially to the scalp, with the handle pointing posteriorly and laterally at an approximate 45° angle from the mid-sagittal line. The optimal scalp position (“motor hotspot”) for eliciting MEPs in the contralateral first dorsal interosseous (FDI) muscle was identified and marked in navigation system. The resting motor threshold (RMT) was defined as the minimum stimulus intensity required to produce MEPs with a peak-to-peak amplitude of at least 50 μV in the relaxed FDI muscle in at least five out of 10 consecutive trials.

For the experimental assessments, the stimulus intensity was set to 115% of the RMT. To evaluate motor cortex excitability dynamics in response to the stressor, MEPs were recorded in three blocks of 15 pulses each. These blocks were administered at the following time points:

1. Pre-stress baseline: immediately before the application of the CPT.
2. Post-stress: immediately following the end of the CPT.
3. Recovery: 15 min after the CPT.

Throughout the TMS procedure, participants were instructed to remain relaxed. Electromyographic (EMG) activity was recorded from the FDI muscle using surface electrodes to ensure muscle relaxation and to capture MEP amplitudes. The mean peak-to-peak MEP amplitude was calculated for each block of 15 trials for subsequent statistical analysis. Correlations between the parameters of the ANS and CNS were of particular interest.

## 3 Statistical analysis

All statistical analyses were performed using IBM SPSS Statistics (Version 28.0). The alpha level for statistical significance was set at *p* < 0.05. Corrections for multiple comparisons (Bonferroni) were applied.

### 3.1 Data normalization and preparation

Prior to inferential analysis, the normality of all continuous variables was assessed using the Shapiro–Wilk test, and the homogeneity of variances was verified using Levene’s test. Based on these assessments, parametric or non-parametric tests were selected to ensure the validity of the results. Change scores (Δ) for all key variables (e.g., MEP amplitude, heart rate, blood pressure, subjective emotional state assessment) were computed as the difference between post-stress and pre-stress measurements to quantify the dynamics of the stress response.

The analysis was structured to evaluate the differential effects of stress type on emotional, physiological, and neurophysiological indices.

### 3.2 Comparative analysis of stress type effects

(a) Emotional Indicators: A 2 (Stress Type: CPT vs. TSST) × 3 (Time: Pre-stress, Post-stress, Recovery) factorial Multivariate Analysis of Variance (MANOVA) was conducted on the emotional state indicators. This method was selected because it allows the simultaneous testing of the effects of the independent variables on a combination of correlated dependent variables, thereby controlling Type I error effects. Pillai’s Trace was used as the multivariate test statistic due to its robustness. Significant main and interaction effects were followed up with post-hoc pairwise comparisons using independent and paired-samples t-tests with a Bonferroni adjustment.
(b) Physiological Indicators: A similar 2 × 3 factorial MANOVA was initially performed for the physiological parameters (SBP, DBP, HR). However, as the data for some measures violated the assumptions of parametric tests, the inter-group differences in response dynamics were further quantified using the non-parametric Mann–Whitney U test for independent samples. This approach provides a more robust comparison when parametric assumptions are not met. The magnitude of these differences was assessed using Z-statistics and Cohen’s *d* for effect size.

### 3.3 Analysis of motor cortical excitability (MEP)

The effect of stress on corticospinal excitability was assessed using a one-way repeated-measures Analysis of Variance (ANOVA) with ‘Time’ as the within-subject factor for MEP amplitude. This design is statistically powerful for detecting within-subject changes over time, as it accounts for inter-individual variability.

To investigate the relationship between central and peripheral stress markers, Pearson correlation coefficients were calculated between the change scores (Δ) for MEP amplitude and the change scores for key cardiovascular parameters (ΔHR, ΔSBP, ΔDBP). A Bonferroni correction was applied.

## 4 Results

### 4.1 Comparative analysis of the effects of stress type

#### 4.1.1 Analysis of emotional indicators

The observed cardiovascular and emotional responses were consistent with the expected effects of stress induction, supporting the validity of the experimental protocols. ANOVA showed a statistically significant main effect of the “Time” factor (*p* = 0.029), indicating substantial changes in emotional indicators between consecutive measurements. This effect was confirmed by all applied multivariate criteria (Pillai’s Trace, Wilks’ Lambda, Hotelling’s Trace, and Roy’s Largest Root), which demonstrated a consistent level of significance (p = 0.029) (Appendix Tables A1, A2).

Of particular interest is the significant interaction effect of “Time × Stress Type” (Pillai’s Trace, *p* = 0.040), indicating the differential influence of various stressors on emotional state.

Post-hoc analysis revealed the following patterns: before stress exposure, the groups did not differ on any of the measures (*p* > 0.05), confirming their initial comparability. A comparison of the groups after stress exposure revealed a significantly higher level of tension in the psychological stress group compared to the physiological stress group (*p* = 0.023).

**Figure 2:**
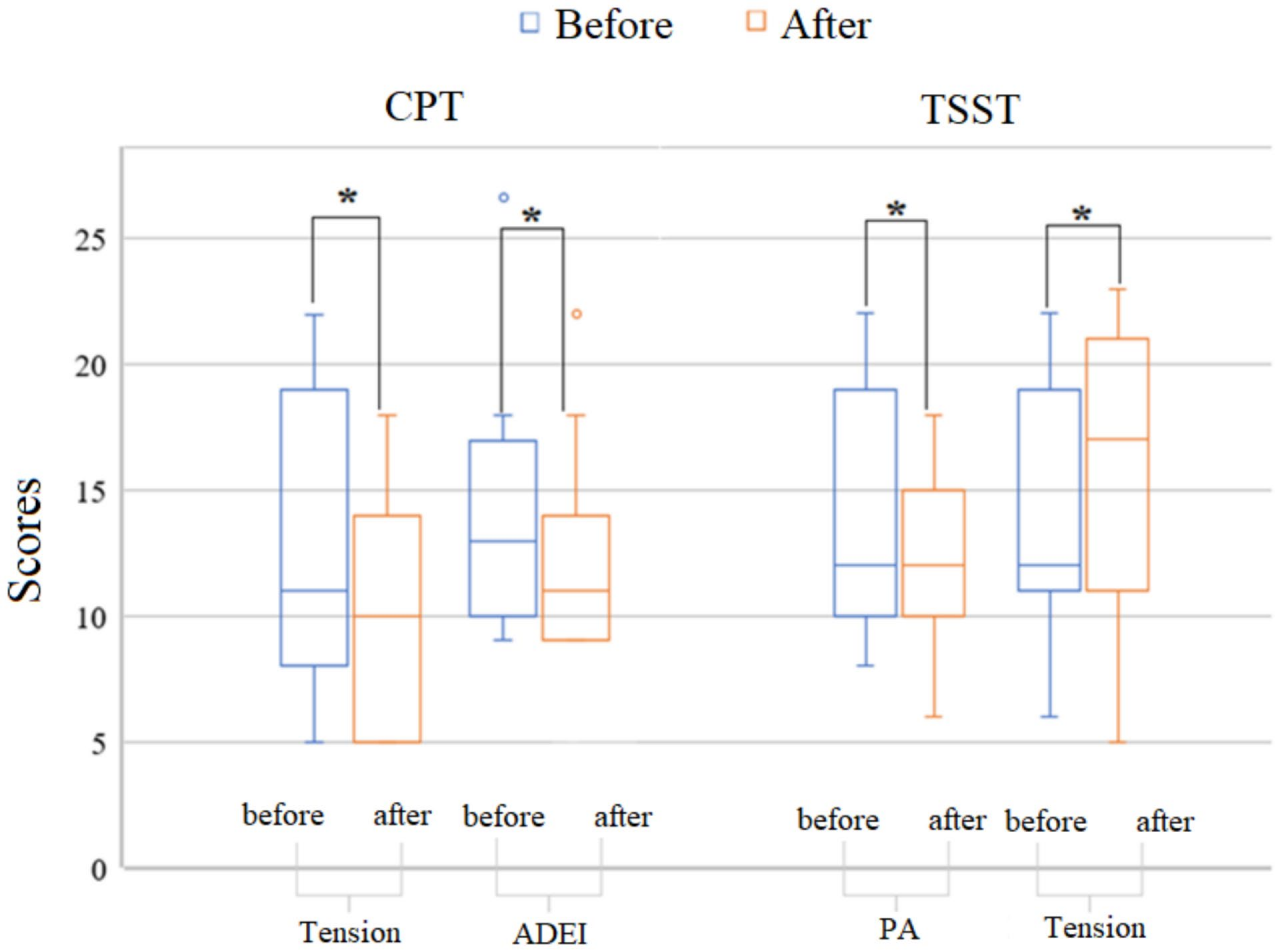
Dynamics of subjective psychological assessment of emotional state indicators. ADEI, Anxiety-Depressive Emotions Index; PA, Positive Affect; (the range of the scales are the same, see methods, paragraph 2.2.2) CPT, Cold Pressor Test (physiological stress); TSST, Trier Social Stress Test (social/psychological stress). The figure shows the group means for the CPT group and the TSST group across three time points: before, immediately after, and 15 min after stress exposure. The ordinate scale shows the scores for the specified variables. *see significant inter-group differences (*p* < 0.05).

In the CPT group, a decrease in the level of tension (*p* = 0.049) and anxious-depressive emotions (*p* = 0.027) was observed immediately after induction. Conversely, in the TSST stress group, an increase in tension (*p* = 0.044) and a decrease in positive affect (*p* = 0.027) were observed (see Figure 2).

#### 4.1.2 Analysis of physiological indicators

MANOVA revealed a significant influence of:

The Type of stress exposure on SBP (*F* = 18.018, *p* < 0.001) and HR (*F* = 28.416, *p* < 0.001), but not on diastolic blood pressure (DBP) (*F* = 1.244, *p* = 0.292). The Time factor on the dynamics of SBP (*F* = 26.032, *p* < 0.001) and HR (*F* = 21.249, *p* < 0.001), with no significant effect on DBP (*F* = 1.084, *p* = 0.341).

The most significant result was the interaction effect “Stress Type × Time” for SBP (*F* = 21.785, *p* < 0.001) and HR (*F* = 39.102, *p* < 0.001), indicating different response dynamics depending on the type of stressor. For DBP, the interaction was not significant (*F* = 0.407, *p* = 0.804).

**Figure 3:**
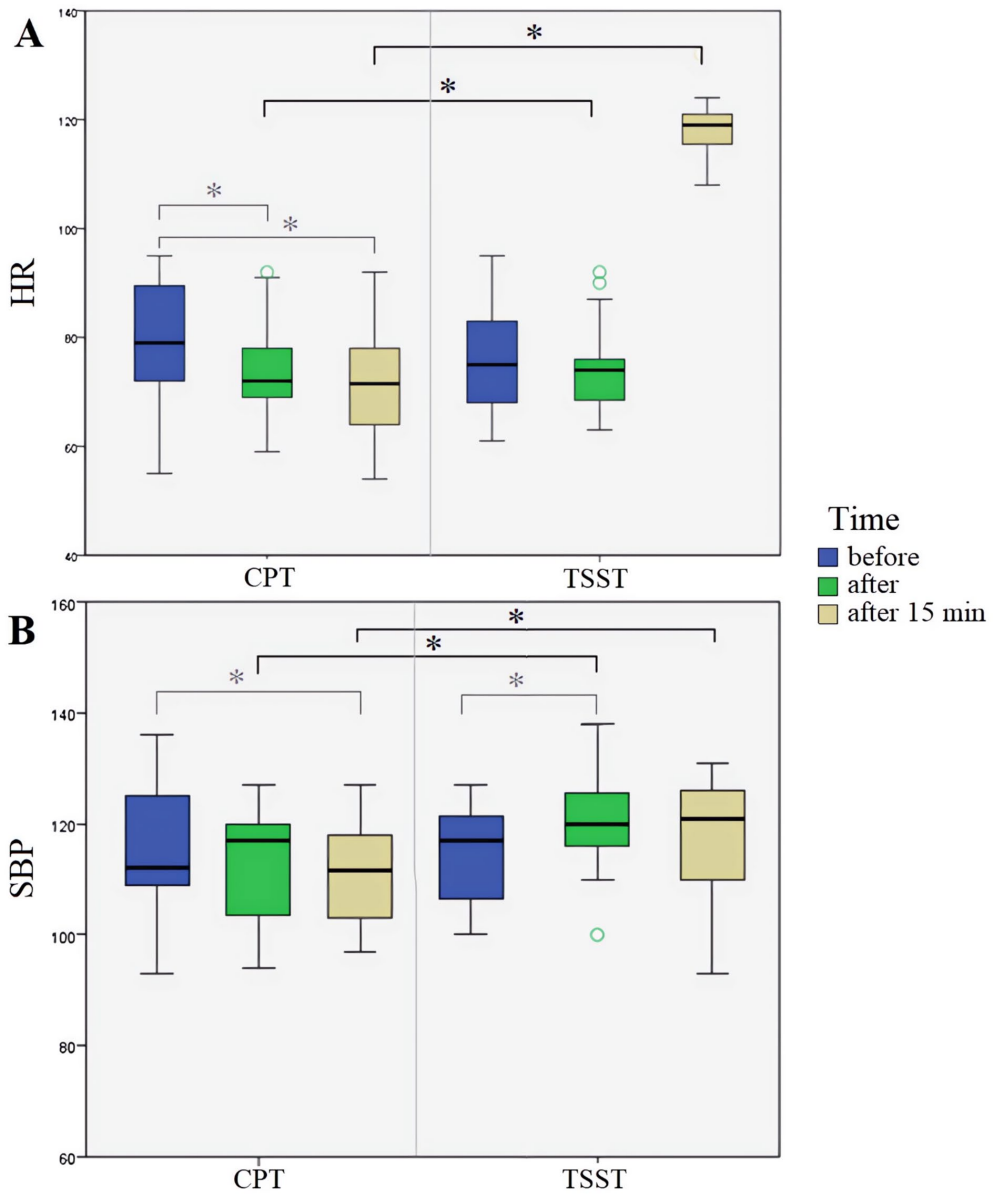
Results of the comparison of measurements taken before, after, and 15 min after stress exposure in the two groups. HR, heart rate; SBP, systolic blood pressure; CPT, Cold Pressor Test; TSST, Trier Social Stress Test. The figure illustrates the group means for the CPT group (left side) and the TSST group (right side) across three time points: before, immediately after, and 15 min after stress exposure. **(A)** Displays the results for HR dynamics. **(B)** Displays the results for SBP dynamics. *Significant differences (*p* < 0.05).

Comparative analysis revealed substantial differences in the dynamics of psychophysiological reactions to different types of stress exposure (see Figure 3). In the psychological stress group, significantly higher SBP and HR levels persisted 15 min after exposure compared to the physiological stress group (*p* < 0.01). In the physiological stress group, a statistically significant decrease in SBP and HR was observed both immediately after the load (*p* < 0.05) and after the 15-min recovery period (*p* < 0.01).

To further quantify the magnitude of intergroup differences in stress response patterns, we conducted additional non-parametric analyses with Z-statistics derived from Mann–Whitney U tests. The results revealed systematic differences in cardiovascular recovery dynamics between stress conditions: The psychological stress group showed significantly higher SBP levels during recovery compared to the physiological stress group (*Z* = −3.42, *p* = 0.001, Cohen’s *d* = 0.78), indicating sustained cardiovascular activation. Similarly, HR remained elevated in the psychological stress condition throughout the recovery period (*Z* = −2.89, *p* = 0.004, Cohen’s *d* = 0.65), demonstrating prolonged autonomic arousal.

#### 4.1.3 Dynamics of primary motor cortical excitability (MEP)

The analysis of MEPs revealed a complex picture of TMS’s modulatory influence on the cortical components of the stress response (Appendix Tables A3, A4).

Analysis of MEP amplitude showed no statistically significant main effect of “Time” factor (*p* = 0.234), indicating that, on average, stress exposure did not induce a systematic, unidirectional change in motor cortex excitability across the entire sample. However, further analysis revealed pronounced individual variability in excitability dynamics that was not fully explained by the type of stressor. This suggests that individuals exhibited divergent response patterns—with some showing increased and others decreased excitability—highlighting the presence of person-specific factors in cortical stress reactivity.

To estimate the relationship between central and peripheral stress markers, we performed Pearson correlation analyses to assess the association between the change scores (post-stress minus pre-stress) for MEP amplitude and key physiological parameters (heart rate, systolic and diastolic blood pressure) across the entire sample. After applying a Bonferroni correction for the three correlations tested (adjusted alpha = 0.017), a significant positive correlation between the dynamics of MEP amplitude and the change in heart rate was confirmed (*r* = 0.581, *p* = 0.047). This result suggests that subjects with a more pronounced increase in HR after stress also exhibited a greater increase in MEP amplitude, implying a functional coupling between motor cortex excitability and autonomic reactions under stress.

The correlation between MEP changes and HR response was further validated using regression analysis (standardized *β* = 0.53, *Z* = 2.67, *p* = 0.008), confirming the strength of the cortico-autonomic coupling. No other correlations with cardiovascular measures reached significance.

## 5 Discussion

### 5.1 Comparative analysis of reactions to psychological and physiological stress

The stress induction was valid, as evidenced by the expected psychophysiological response pattern. Participants exhibited significant changes in heart rate and blood pressure, coupled with self-reports of increased tension and high subjective stress ratings on a 5-point scale. The average rating for both groups was 4.4.

The present study revealed fundamental differences in the dynamics of physiological indicators in response to different types of stress exposure. The TSST produced more enduring changes in systolic blood pressure and heart rate, which persisted for 15 min post-exposure. In contrast, the CPT led to a rapid normalization of these parameters, consistent with the current understanding of stress response specificity (Lamotte et al., 2021b). Of particular interest was the identified difference in HR dynamics: psychological stress was associated with a prolonged increase in HR, which persisted 15 min post-exposure, whereas physiological stress resulted in a rapid normalization of this measure. A similar pattern emerged for systolic blood pressure: psychological stress caused a sustained elevation, while physiological stress produced a biphasic response—an initial increase followed by a compensatory decrease.

Numerous studies confirm that psychological and physiological stressors activate distinct physiological adaptation mechanisms (James et al., 2023). The prolonged cardiovascular activation characteristic of psychological stress is well-established and largely attributed to sustained neuroendocrine activity involving the sympatho-adrenal medullary (SAM) system and the hypothalamic–pituitary–adrenal (HPA) axis. This activity leads to continued release of catecholamines and cortisol (Dimsdale, 2008). In contrast, physical stressors typically result in a more transient response involving a complex interplay of sympathetic and parasympathetic influences (Moses et al., 2023).

While this general neuroendocrine framework is recognized, the central mechanisms and dynamic interplay between different physiological systems that govern these distinct temporal profiles remain poorly understood. One critical, unresolved question is how the brain coordinates these system-wide responses. Sherrington’s “funnel” principle can be used to conceptualize this process. According to this principle, a multitude of supraspinal inputs, including cognitive, emotional, and homeostatic signals, converge to produce unified somatic and ANS outputs (Grillner and El Manira, 2020; Kandel et al., 2021). However, it is unclear how central nervous system activity, particularly in regions such as the motor cortex—a key supraspinal integrator of cognitive and motor commands that projects to the spinal final common pathway—co-varies with peripheral dynamics. The protracted recovery profile of psychosocial stress may reflect the sustained convergence of limbic and cognitive inputs into this funnel. In contrast, the rapid normalization after physical stress may represent the quicker dissipation of a more localized homeostatic signal (LeDoux and Pine, 2016).

This discrepancy may reflect the organism’s distinct adaptation strategies: mobilizing resources in response to social threat versus localized vascular adaptation during physical stress. These phenomena could be explained by different mechanisms of sympathetic nervous system activation: catecholamine release during social stress versus localized vascular reactions during cold exposure (Han et al., 2022).

Psychological stress (e.g., taking an exam or speaking in public) activates the sympathetic nervous system, leading to a sustained increase in HR and SBP, even 15 min after exposure (Kukso and Kukso, 2022). This is associated with prolonged adrenaline and cortisol release, which maintain vascular tone and cardiac activity. This effect is further amplified by persistent cognitive rumination on the stressful event, which continues to activate central stress circuits even in the absence of immediate threat (Zoccola et al., 2008). Physical stress (e.g., exercise) causes a sharp rise in HR and SBP during the load. After its cessation, however, parasympathetic mechanisms are activated, resulting in vasodilation and a rapid decrease in blood pressure. After 15 min, these parameters often return to normal or drop below baseline due to post-exercise hypotension (Ginty et al., 2022). During psychological stress, prolonged sympathetic activation dominates as the organism continues to “anticipate” a threat. In physical stress, compensatory mechanisms (e.g., baroreflexes) quickly engage to normalize pressure after the load (Nemets and Vinogradova, 2017).

Another important question is the discrepancy between objective physiological indicators of stress and subjective assessments of emotional state. Our data demonstrated the complex nature of this relationship. While physiological stress was associated with an objective decrease in cardiovascular activity accompanied by a reduction in subjectively rated tension, psychological stress caused the opposite dynamic: an increase in ANS activation, a rise in subjective tension, and a decrease in positive affect.

These findings are consistent with the concept of distinct adaptation strategies in organisms: mobilizing psychoemotional resources in response to social threats (Kemeny, 2009) as opposed to localized vascular reactions during physical stress (Plante, 2002).

This may indicate a dissociation between the subjective perception of stress and objective physiological shifts that requires further investigation. Are the subjective perception of stress and ANS reactions regulated by different mechanisms? This effect may be difficult to observe because subjective stress perception depends on individual coping strategies and cognitive appraisals rather than solely on physiological changes (Nemets and Vinogradova, 2017). This discrepancy could be explained by different neurobiological mechanisms for processing various stressor types. If physical stress primarily activates peripheral regulatory systems, then psychosocial stress involves higher cognitive and emotional structures. Overall, this result contradicts previous studies that suggested a direct link (Villanueva et al., 2016) and emphasizes the importance of individual coping strategies and cognitive biases in self-assessment. Studies on academic stress, a specific form of chronic psychosocial stress arising from educational demands such as exams, deadlines, and pressure to perform well academically, have also revealed a discrepancy between subjective reports and objective measures such as electrodermal activity (EDA) and cortisol levels (Villanueva et al., 2016). However, the present work is the first to systematically analyze this dissonance in relation to different stressor types.

Furthermore, the obtained results partially diverge from the data of other studies that reported more pronounced reactions to physiological stress (Han et al., 2022). This discrepancy may be related to methodological specifics, such as water temperature and exposure duration, or sample characteristics, underscoring the need to standardize stress-testing protocols (Lamotte et al., 2021a).

The findings allowed us to interpret the discovered differences through the lens of different activated physiological mechanisms. Reactions sustained under psychological stress are linked to prolonged activation of the sympathetic nervous system, which manifests as persistent increases in cardiovascular activity and peripheral vasoconstriction. This reaction is characteristic of states that require the prolonged mobilization of resources in response to social threats.

In contrast, reactions to physiological stress demonstrate a different dynamic, reflecting a more localized and short-term adaptation. The rapid normalization of ANS balance in this case may be due to the specific nature of physiological stress. Unlike psychosocial stress, physiological stress does not require maintaining a state of prolonged readiness. However, data showing a decrease in stress indicators after physiological exposure contradict the results of other studies demonstrating sustained reactions to CPT (Han et al., 2022; Lamotte et al., 2021a).

Notably, the identified physiological differences align well with the dynamics of subjective emotional experiences. Maintaining elevated tension after psychological stress and reducing it after physiological exposure supports the concept of distinct organismal adaptation strategies depending on the nature of the stressor. These differences may be associated with the involvement of different neurobiological systems. Physical stress primarily activates peripheral regulatory mechanisms, while psychosocial stress engages complex interactions between the cortex and limbic system (James et al., 2023).

### 5.2 Motor cortex excitability and рeripheral responses under stress

While our exploratory analysis suggests a potential link, the current data do not provide conclusive evidence that autonomic stress patterns can be reliably quantified via primary motor cortex excitability changes, as no systematic group-level MEP modulation was found.

The data on the dynamics of primary motor cortical excitability, assessed using single-pulse TMS, reveal complex changes in cortical processes under stress. We understand that the TMS method tests the excitability not only of the motor cortex but also of the entire corticospinal system. However, the excitability of the motor cortex plays a significant role in evoked motor responses (Lefaucheur, 2019). Based on literature (Al et al., 2023), we suggest the observed effects are specifically associated with primary motor cortex activity. The absence of a statistically significant main effect of “time” indicates that, on average across the sample, stress exposure did not induce a unidirectional, systematic change in motor cortex excitability. This suggests that the motor cortex exhibits a degree of functional stability to the stressor at the population level (Gellner et al., 2022). However, the highly significant “Subject × Time” interaction effect refines this interpretation, indicating substantial response heterogeneity. This means that, in response to stress, different participants showed divergent, individualized changes in excitability ranging from enhancement to suppression, which were masked in the group-level analysis. This is consistent with known individual differences in stress vulnerability and ANS reactivity (Gianaros and Wager, 2015). However, our results contrast with studies reporting general cortical inhibition under stress. These discrepancies may reflect methodological differences in stress paradigms or a focus on prefrontal rather than motor regions (Al et al., 2020). Despite a sample size comparable to field standards, the absence of a uniform group-level change in MEP excitability in our study robustly highlights that stress-induced cortical changes are highly specific to the neural circuit and individual physiological response profile, not monolithic.

The correlations between the parameters of the ANS and CNS are of particular interest. The most significant finding is the positive correlation detected between MEP amplitude dynamics and changes in heart rate. This finding is crucial for understanding the integrative mechanisms of the stress response. It enables us to hypothesize the existence of a close functional link between central motor systems and peripheral manifestations of stress and demonstrates a close connection between higher motor representations and the effects of stress. Specifically, this finding builds upon the work of Al et al. (2023), who demonstrated that cardiac activity directly modulates motor cortical excitability. It shows that this relationship dynamically manifests during psychological stress (Al et al., 2023; Tanaka et al., 2025). The discovered covariance suggests that the strength of an individual’s ANS stress response is associated with the degree to which their motor cortex excitability changes (Grosprêtre et al., 2025). This may indicate shared underlying neurophysiological mechanisms that coordinate higher nervous activity and the somatic and autonomic components of the reaction (Thayer and Lane, 2009). One likely candidate is the generalized activation of descending pathways from limbic structures (e.g., the amygdala) and the brainstem (Benarroch, 1993; LeDoux and Pine, 2016), which influence sympathetic tone (Beissner et al., 2013) and cortical excitability (Sabino-Carvalho et al., 2021) simultaneously, preparing the organism for a “fight-or-flight” response (Cannon, 1967). This integrated preparation likely involves enhancing motor readiness in tandem with cardiovascular activation (Al et al., 2023).

These findings consequently underscore the necessity of considering the stress response as a holistic organismic state. Using TMS as a probe, we demonstrated that stress-induced changes in peripheral physiology are tightly integrated with the dynamics of cortical structures involved in motor preparation. Although physiological links between cardiovascular function and cortical excitability exist at rest (e.g., Al et al., 2023), the specific, coordinated increase in heart rate and motor cortex excitability observed here suggests a mechanism unique to the stress response. These results suggest that, during stress, shared ascending pathways from the brainstem and limbic structures are activated. These pathways simultaneously drive sympathetic arousal and a state of heightened motor readiness. This creates a unified psychophysiological state of preparedness for action that differs from baseline cardiovascular-cortical interactions. The observed variability in MEP responses indicates that the cognitive-motor interface during stress is not uniform but rather a key source of individual differences in stress reactivity. Thus, our data positively answer a key question in the field by demonstrating that an individual’s characteristic pattern of stress response can be quantified by assessing motor cortical activation dynamics.

Future research should assess whether these distinct excitability patterns predict behavioral outcomes, such as motor performance or decision-making abilities, in stressful situations.

### 5.3 Limitation

The modest sample size and recruitment from a specific population (university students) limit the generalizability of our findings. Furthermore, the observed inter-individual variability in stress responses may be partially attributed to unaccounted sex-specific reactivity patterns, which future studies should control for. A notable limitation of this study is the absence of precise randomization or stratification of female participants based on their menstrual cycle phase, which is known to modulate neuroendocrine stress reactivity via fluctuations in estradiol and progesterone levels (Zhu et al., 2016; Dobson et al., 2026). Future investigations should rigorously control hormonal status by either scheduling experimental sessions according to specific menstrual cycle phases or by directly measuring serum levels of key steroid hormones to account for this significant source of variance in stress responses.

## 6 Conclusion

This study revealed significant differences in psychophysiological responses to physical and psychological stress. Physical stress caused a temporary increase in cardiovascular activity, which was quickly followed by normalization of the parameters. This was accompanied by a reduction in subjective tension and anxiety. In contrast, psychological stress resulted in prolonged elevations in blood pressure and heart rate that persisted into the recovery phase. This was accompanied by increased subjective tension and diminished positive affect.

These findings suggest that there are distinct adaptation mechanisms to stressors of different natures. Physical stress primarily activates peripheral regulatory mechanisms that enable the swift restoration of homeostasis. In contrast, psychological stress involves complex cortico-limbic interactions that underlie its protracted response. These results highlight the importance of a differentiated approach to assessing stress reactions and developing stress management interventions tailored to the specific characteristics of the stressor.

Furthermore, this study uses TMS to probe cortical excitability and provides novel evidence of a direct link between central motor and peripheral autonomic processes under stress. The positive correlation identified between MEP dynamics and heart rate changes highlights the integrative nature of the stress response. This suggests that there are shared neurophysiological mechanisms that coordinate somatic and autonomic reactivity. The significant variability in cortical excitability among individuals emphasizes the importance of considering person-specific response patterns in stress research.

Consequently, using TMS as a measurement tool is a valuable approach for multi-system stress assessments, capturing the interplay between cortical and autonomic states. Our findings demonstrate that characteristic patterns of the autonomic stress response can be effectively measured and quantified through associated changes in primary motor cortical excitability. Future research should leverage these integrative methodologies to further elucidate the neural substrates of stress vulnerability and resilience.

## Data Availability

The raw data supporting the conclusions of this article will be made available by the authors without undue reservation.
