# Supplementary material for: Social and physiological stress elicit divergent psycho-physiological dynamics and motor cortex activation
Source: Front Psychol. 2026 Mar 5;17:1760772. doi: 10.3389/fpsyg.2026.1760772 (PMC12999422; doi:10.3389/fpsyg.2026.1760772)
Supplement: Supplementary file 1 [file Table_1.DOCX]

**Appendix A**

**Table A1.** Results of analysis of variance (ANOVA) of the influence of stress type and time on physiological parameters

| Source | Dependent Variable | Type III Sum of Squares | df | Mean Square | F | Sig. |
| --- | --- | --- | --- | --- | --- | --- |
| Stress_Type | Systolic Pressure | 3718.711 | 2 | 1859.356 | 18.018 | .000 |
|  | Diastolic Pressure | 254.163 | 2 | 127.082 | 1.244 | .292 |
|  | Heart Rate | 5395.859 | 2 | 2697.930 | 28.416 | .000 |
| Time | Systolic Pressure | 5372.819 | 2 | 2686.410 | 26.032 | .000 |
|  | Diastolic Pressure | 221.363 | 2 | 110.682 | 1.084 | .341 |
|  | Heart Rate | 4034.892 | 2 | 2017.446 | 21.249 | .000 |
| Stress_Type * Time | Systolic Pressure | 8992.403 | 4 | 2248.101 | 21.785 | .000 |
|  | Diastolic Pressure | 166.072 | 4 | 41.518 | 0.407 | .804 |
|  | Heart Rate | 14850.116 | 4 | 3712.529 | 39.102 | .000 |

**Table A2.** Multivariate analysis (MANOVA) of the influence of stress type and time on psychological indicators (self-assessment of emotional states)

| Effect | | Value | F | Hyp df | Error df | Sig. |
| --- | --- | --- | --- | --- | --- | --- |
| **Between Groups** | | | | | | |
| Intercept (Pillai's Trace) | 0.999 | | 1302.657 | 16.000 | 27.000 | .000 |
| Intercept (Wilks' Lambda) | 0.001 | | 1302.657 | 16.000 | 27.000 | .000 |
| Intercept (Hotelling's Trace) | 771.945 | | 1302.657 | 16.000 | 27.000 | .000 |
| Intercept (Roy's Largest Root) | 771.945 | | 1302.657 | 16.000 | 27.000 | .000 |
| Group (Pillai's Trace) | 0.785 | | 1.130 | 32.000 | 56.000 | .338 |
| Group (Wilks' Lambda) | 0.365 | | 1.105 | 32.000 | 54.000 | .365 |
| Group (Hotelling's Trace) | 1.329 | | 1.080 | 32.000 | 52.000 | .395 |
| Group (Roy's Largest Root) | 0.840 | | 1.471 | 16.000 | 28.000 | .181 |
| **Within Groups** | | | | | | |
| Time_of_Assessment (Pillai's Trace) | 0.573 | | 2.267 | 16.000 | 27.000 | .029 |
| Time_of_Assessment (Wilks' Lambda) | 0.427 | | 2.267 | 16.000 | 27.000 | .029 |
| Time_of_Assessment (Hotelling's Trace) | 1.344 | | 2.267 | 16.000 | 27.000 | .029 |
| Time_of_Assessment (Roy's Largest Root) | 1.344 | | 2.267 | 16.000 | 27.000 | .029 |
| Time*Stress_Type (Pillai's Trace) | 0.986 | | 1.701 | 32.000 | 56.000 | .040 |
| Time*Stress_Type (Wilks' Lambda) | 0.256 | | 1.646 | 32.000 | 54.000 | .052 |
| Time*Stress_Type (Hotelling's Trace) | 1.957 | | 1.590 | 32.000 | 52.000 | .067 |
| Time*Stress_Type (Roy's Largest Root) | 1.095 | | 1.917 | 16.000 | 28.00 |  |

**Table A3.** Results of Mixed ANOVA for MEP Amplitude with Subject and Time Factors. Dependent Variable: MEP

| Source | Type III Sum of Squares | df | Mean Square | F | Sig. |
| --- | --- | --- | --- | --- | --- |
| Corrected Model | 5.825a | 11 | .530 | 2.621 | .079 |
| Intercept | 1.553 | 1 | 1.553 | 7.683 | .022 |
| time | .028 | 1 | .028 | .138 | .719 |
| resp | 5.787 | 10 | .579 | 2.864 | .064 |
| Error | 1.819 | 9 | .202 |  |  |
| Total | 20.952 | 21 |  |  |  |
| Corrected Total | 7.643 | 20 |  |  |  |

a. R Squared = .762 (Adjusted R Squared = .471)

**Table A4.** Results of Mixed ANOVA for MEP Amplitude with Subject × Time Interaction. Dependent Variable: MEP

| Source | Type III Sum of Squares | df | Mean Square | F | Sig. |  |
| --- | --- | --- | --- | --- | --- | --- |
| Corrected Model | 166,170,167,163.686ᵃ | 35 | 4,747,719,061.820 | 7.370 | .000 | |
| Intercept | 187,339,851,580.955 | 1 | 187,339,851,580.955 | 290.798 | .000 | |
| respondent | 93,521,921,295.369 | 11 | 8,501,992,845.034 | 13.197 | .000 | |
| time | 1,880,409,923.875 | 2 | 940,204,961.938 | 1.459 | .234 | |
| respondent * time | 70,332,904,420.099 | 22 | 3,196,950,200.914 | 4.962 | .000 | |
| Error | 228,700,787,368.674 | 355 | 644,227,570.053 |  |  | |
| Total | 642,776,617,823.500 | 391 |  |  |  | |

ᵃ R Squared = .421 (Adjusted R Squared = .364)
